# A High-Fructose-High-Coconut Oil Diet Induces Dysregulating Expressions of Hippocampal Leptin and Stearoyl-CoA Desaturase, and Spatial Memory Deficits in Rats

**DOI:** 10.3390/nu9060619

**Published:** 2017-06-16

**Authors:** Ching-I Lin, Chu-Fu Shen, Tsui-Han Hsu, Shyh-Hsiang Lin

**Affiliations:** 1 Department of Nutrition and Health Sciences, Kainan University, No. 1, Kainan Rd., Luchu, Taoyuan 338, Taiwan; cilin@mail.knu.edu.tw; 2 School of Nutrition and Health Sciences, Taipei Medical University, No. 250, Wu-Hsing Street, Taipei 110, Taiwan; m507100012@tmu.edu.tw (C.-F.S.); m507100015@tmu.edu.tw (T.-H.H.); 3 Research Center of Geriatric Nutrition, Taipei Medical University, No. 250, Wu-Hsing Street, Taipei 110, Taiwan

**Keywords:** fructose, fat, leptin, stearoyl-CoA desaturase, coconut, soybean

## Abstract

We investigated the effects of high-fructose-high-fat diets with different fat compositions on metabolic parameters, hippocampal-dependent cognitive function, and brain leptin (as well as stearoyl-CoA desaturase (SCD1) mRNA expressions). Thirty-two male Wistar rats were divided into 3 groups, a control group (*n* = 8), a high-fructose soybean oil group (37.5% of fat calories, *n* = 12), and a high-fructose coconut oil group (37.5% of fat calories, *n* = 12) for 20 weeks. By the end of the study, the coconut oil group exhibited significantly higher serum fasting glucose, fructosamine, insulin, leptin, and triglyceride levels compared to those of the control and soybean oil groups. However, hippocampal leptin expression and leptin receptor mRNA levels were significantly lower, while SCD1 mRNA was significantly higher in rats fed the high-fructose-high-coconut oil diet than in rats fed the other experimental diets. In addition, the coconut oil group spent significantly less time in the target quadrant on the probe test in the Morris water maze (MWM) task. Rats fed the high-fructose-high-coconut oil diet for 20 weeks were prone to develop hyperglycemia, hyperinsulinemia, hyperleptinemia, and hypertriglyceridemia. These metabolic consequences may contribute to hippocampal-dependent memory impairment, accompanied by a lower central leptin level, and a higher SCD1 gene expression in the brain.

## 1. Introduction

Environmental factors such as diet (i.e., excessive caloric intake) significantly contribute to the causation of chronic diseases including obesity and type 2 diabetes mellitus (T2DM) [1]. Individuals with obesity and/or T2DM may place themselves at an increased risk of developing memory deterioration and cognitive impairment, and, in turn, neurodegenerative diseases [2,3]. A dietary pattern which is similar to the typical Western diet (characterized by a high intake of meat, butter, high-fat dairy products, eggs, and refined sugar) was demonstrated to predict an increased risk of neurodegenerative disease, i.e., Alzheimer’s disease (AD) [4]. Among the components of a Western diet, high intakes of saturated fats and simple carbohydrates (e.g., refined sugar) have garnered much attention for their roles in the development of cognitive dysfunction and neurodegenerative diseases [5,6]. Animal studies suggest that a high-fat diet rich in saturated fatty acids (SFAs) can result in obesity as well as deficits in hippocampal-dependent learning and memory processes [7,8]. A high-fructose diet alone has also been implicated in not only promoting obesity and insulin resistance but also cognitive impairments involving neurodegenerative diseases [9,10]. However, the impacts of the combination of a high-fat and high-fructose diet on the hippocampal-dependent cognitive function are less well understood. It is conceivable that accentuated metabolic disorders can occur due to the synergetic effects of a combined high fat and high fructose diet. There is evidence that rats fed a high-fructose-high-fat diet develop metabolic disorders characteristic of the metabolic syndrome (MetS) more severely than a high fat diet or high fructose diet alone [11]. Despite epidemiological evidence of a link between MetS and cognitive decline [12], it is not known whether the metabolic consequences imposed by a high-fructose-high-fat diet are also likely to impact hippocampal-dependent cognitive function. Evidence indicates that cognitive function is differentially affected by saturated and unsaturated fat [4]. It should be noted that although high-fructose-high-fat diets have been demonstrated to be effective in developing MetS-associated complications [11], these diets are usually formulated by mixing various types (i.e., animal- and plant-based fats) and amounts of fats [13,14,15,16,17]. To the best of our knowledge, there have been no studies in which the effects of high-fructose-high-fat diets with two different types of plant-based oil on changes of the metabolism and hippocampal-dependent cognition were compared.

Furthermore, evidence from rodent studies has proven that central and peripheral leptin resistance is the consequence of a high-fructose-high-fat diet that increases obesity susceptibility [18]. Interestingly, central leptin resistance due to impaired regulation of leptin-mediated signaling has also been implicated in Alzheimer-type neurodegeneration [19]. Therefore, it is reasonable to assume that leptin and its mediated signaling may have pathogenic roles in diet-related metabolic disorders as well as neurodegeneration [19,20]. Notably, stearoyl-CoA desaturase (SCD) is regarded as a component of leptin signaling and is also known as Δ9 desaturase, which participates in the biosynthesis of monounsaturated fatty acids (MUFA) [21]. This enzyme converts the SFA CoAs, palmiatoyl-CoA(16:0), and stearoyl-CoA (18:0) into the MUFA CoAs, palmiatoleyl-CoA (16:1 n7), and oleyl-CoA (18:1, n9), respectively [21]. In SCD null mice, it was observed that diet-induced or leptin deficiency-induced obesity was prevented due to reduced body adiposity and improved insulin sensitivity [22,23], suggesting SCD as a potential therapeutic target for metabolic complications associated with obesity [23]. In addition to obesity, SCD action may also involve the development of Alzheimer-type neurodegeneration at molecular levels, as evident by the increased expression of SCD mRNA in subjects with AD [24]. Moreover, increased SCD mRNA expression appeared to be well correlated with impaired cognition functioning [24]. 

Taken together, we hypothesized that aberrant metabolic processes and impaired central leptin-mediated signaling might share pathways for the effects of a high-fructose-high-fat diet and hippocampal-dependent cognitive deficiency which might further trigger the development of neurodegeneration. The aim of the present study was to investigate the effects of different fats (more unsaturated vs. more saturated, based on a high-fructose-high-fat condition) on metabolic parameters, hippocampal-dependent cognitive functions, brain leptin, and stearoyl-CoA desaturase (SCD1) mRNA expressions in rats.

## 2. Materials and Methods

### 2.1. Animals

Male Wistar rats (6 weeks old, approximately 200 g in body weight (BW))—obtained from BioLASCO (Yilan, Taiwan)—were housed in the animal facility at Taipei Medical University (TMU) at 23 ± 2 °C, 50~60% relative humidity, and a 12-h light-dark cycle. The rats were allowed access to a standard chow diet (product 1326, Altromin, Lage, Germany) and water ad libitum. Before the experiments, the rats were acclimatized to the environment and diet for two weeks. The animal protocols used in this study were approved by the Institutional Animal Care and Use Committee at Taipei Medical University.

After acclimatization for a period of two weeks, the rats (*n* = 32) were randomly divided into 3 groups subjected to different diets: AIN-93M control diet (control, *n* = 8), high-fructose-high-soybean oil diet (soybean oil, *n* = 12), or high-fructose-high-coconut oil diet (coconut oil, *n* = 12), for 20 weeks. The nutrient composition in the AIN-93M control diet was as follows: 46.6% corn starch, 15.5% dextrin, 10% sucrose, 5% cellulose, 14% casein, 4% soybean oil, 3.5% AIN mineral mix, 1% AIN vitamin mix, 0.25% choline, 0.18% cysteine, 0.2% choline, and 0.0008% tertiary butylhydroquinone. The AIN-93M control diet had an energy density of 3.8 kcal/g and contained 75.8% carbohydrates (mostly from corn starch), 14.7% protein from casein, and 9.5% fat from soybean oil. The experimental (high-fructose-high-fat) diets were similar except that the corn starch, dextrin, sucrose, and soybean oil were replaced by 50.8% fructose and 37.5% fat. Both experimental diets had an energy density of 4.8 kcal/g and contained 50.8% carbohydrates from fructose, 11.7% protein from casein, and 37.5% fat from soybean oil or coconut oil as an abundant source of PUFAs or SFAs, respectively. Food consumption was estimated by weighing the food containers before and after the food was given to the animals. The average consumption was taken for each group to minimize the variation in consumption recorded due to spillage. Food consumption was recorded daily for the first eight weeks and body weight was measured at the beginning and end of the study.

### 2.2. Morris Water Maze (MWM) Task 

The MWM task was performed during four consecutive days following 20 weeks of the experimental diets to evaluate the cognitive function of the rats. The experimental procedure was similar to that described by Morris [25] with some modifications [26]. This task consists of spatial acquisition and probe trials allowing an evaluation of spatial learning and memory. In brief, a circular water tank (150 cm in diameter and 60 cm deep) was used as a test chamber, filled with water to a depth of 30 cm, and maintained at 23 ± 2 °C. It is surrounded by visual cues visible to the rats. A submerged platform (10 cm diameter) was placed 2 cm below the water surface, located in one of the four imaginary quadrants of the tank, and maintained in the same position during all trials. Each rat was given one session of four trials per day for three consecutive days with an inter-trial interval of 10 min and an inter-session interval of 24 h. During each trial, a rat was gently placed into the tank in one of the four quadrants with its face toward the maze, and was allowed 120 s to locate the submersed platform. The location of the platform was kept constant in the acquisition trials. If a rat failed to find the platform in 120 s, it was gently guided to it. Once on the platform, the animal was allowed to remain there for 15 s. At the end of each trial, the rat was towel-dried and returned to its home cage. During each trial, scores for latency to find the platform and the distance traveled from the starting point to the platform were computed and analyzed with image tracking software (FG34PATH, HaSoTec, Rostock, Germany). To assess the reference memory of the rats, a single probe trial was conducted 24 h after the last acquisition day (i.e., on the fourth day). In this trial, the platform was removed from the tank, and a rat was placed in the tank in the quadrant located diametrically opposite the original platform position for 60 s. The distance and time spent in the target quadrant were determined and calculated as an indicator of spatial memory retention.

### 2.3. Blood Biochemical Analysis, and Fasting Serum Insulin, Fructosamine, and Leptin Levels

Blood samples from overnight-fasted rats were collected from the tail vein at the following time points: weeks 0, 4, 8, 12, and 16, and at the end of the MWM task (week 20). Blood samples were allowed to clot at room temperature before centrifugation for 10 min at 4 °C and 3000 rpm, and then the obtained serum was stored at −30 °C. Blood glucose was measured with the hexokinase method. Blood TG, total cholesterol (TC), and low- (LDL) and high-density lipoprotein (HDL) were measured with automated biochemistry equipment. Fasting serum insulin, fructosamine, and leptin levels were determined by means of enzyme-linked immunosorbent assay (ELISA) kits (Mercodia rat insulin kit, Cat. No. 10-1250-01, Mercodia, Uppsala, Sweden; Fructosamine ELISA Kit, ABIN1133744, Atlanta, GA, USA; and Biovendor Mouse/Rat Leptin Kit, Biovendor, Brno, Czech Republic, respectively).

### 2.4. Collection of Tissues

After the cognitive test (MWM) was performed and BWs were recorded, the animals were anesthetized with an intraperitoneal (i.p.) injection of a mixture of Zoletil 50 (Virbac, Carros CEDEX, France) and 2% Rompun (Bayer, Leverkusen, Germany) (1:1 (*v*/*v*), 0.1 mL/kg BW) and sacrificed. They were then transcardially perfused with ice-cold 0.1 M phosphate-buffered saline (PBS, pH 7.4). Following perfusion, the abdominal fat, epididymal fat, and brain were rapidly dissected and weighed. The brain hippocampus was collected [27], snap-frozen in liquid nitrogen, and stored at −80 °C until analyzed. Total body fat was calculated as the sum of the weight of abdominal and epididymal fat pads and relative total body fat weight (the ratio of total body fat weight to final body weight) was used as the adiposity index.

### 2.5. Fatty Acid Analysis of the Brain

Frozen brain tissue samples (100 mg) were homogenized in 3 ml of chloroform-methanol (2:1 *v*/*v*) and lipids were extracted according to Folch et al. [28]. After centrifugation the organic layers were collected and the solvent was evaporated to dryness in a high-vacuum pump. Next, a mixture of borontrifluoride (BF3)/methanol was added to the dry residue and formed fatty acid methyl esters (FAMEs), allowing determination of the fatty acid composition of the lipids. FAMEs were identified and quantified by capillary gas chromatography (GC), equipped with flame-ionization detection (FID) (Thermo Quest, San Jose, CA, USA). An Rtx^®^-2330 capillary column from Restek (cat. no. 10724, Bellefonte, PA, USA), 30 m × 0.32 mm I.D., 0.32-μm film thickness was used. Nitrogen was used as the carrier gas (2 mL/min) with split injection (20:1). Analyses were performed in a programmed temperature mode from 160 °C at 5 °C/min to 250 °C, and then 1 °C/min to 251 °C for 5 min. The detector and injector temperature was 260 °C. The chromatographic data were processed with the Chorm-Card software.

### 2.6. Western Blot Analysis for Brain Leptin

Frozen brain hippocampal samples were lysed with an RIPA buffer and centrifuged at 1500 rpm for 10 min. Protein concentrations were measured using a BCA protein assay kit (Sigma, St. Louis, MO, USA), and protein samples (25 μg) were separated by 10% sodium dodecylsulfate polyacrylamide gel electrophoresis (SDS-PAGE), transferred to polyvinyl difluoride (PVDF) membranes, and blocked with 5% non-fat milk in Tris-buffered saline, Tween-20 (TBST) buffer. Membranes were incubated for 8 h at 4 °C with primary antibodies: rabbit polyclonal anti-Ob (1:200; Santa Cruz Biotechnology, Santa Cruz, CA, USA), and mouse monoclonal anti-β actin (1:1000; Santa Cruz Biotechnology). Membranes were subsequently incubated with anti-rabbit or anti-mouse antibodies for 1 h at room temperature and then reacted with enhanced chemiluminescence reagents. Signals were detected by the UVP Biospectrum AC System (UVP, Upland, CA, USA) and analyzed using the Image-pro Plus software.

### 2.7. Real-Time Reverse-Transcription Polymerase Chain Reaction (RT-PCR) for Leptin Receptor and SCD1 Genes

Total RNA was isolated from the rat brain hippocampus using the Trizol reagent (Invitrogen, Carlsbad, CA, USA) according to the manufacturer’s instructions. Total RNA was spectrophotometrically quantified by measuring the absorbance at 260 nm (A260). Isolated RNA was reverse-transcribed using a Thermo-XTM kit containing oligo dT primers and Thermo-XTM reverse-transcriptase (Invitrogen) to synthesize complementary (c)DNA. The levels of the rat leptin receptor (SCD1) and GAPDH (as the internal control) mRNAs were quantified using SYBR^®^ Green Real-Time PCR Master Mixes on an ABI Prism^®^ 7300 Sequence Detection System (Applied Biosystems, Foster City, CA, USA). The following PCR primers were used: leptin receptor: forward 5′-AAGCATCGTACTGCCCACAA-3′ and reverse 5′-GGAGGCACCGATGGAATTGA-3′; SCD1: forward 5′-TGGTGCTCTTTCCCTGTTTGC-3′ and reverse 5′-TGGGCTTTGGAAGGTGGACA-3′; and GAPDH: forward 5′-CCAGCCCAGCAAGGATACTG-3′ and reverse 5′-CCCCTCCTGTTGTTATGGGG-3′. Multiples of change in the mRNA levels of the leptin receptor and SCD1 were evaluated after being normalized to the expression level of the internal control (GAPDH).

### 2.8. Statistical Analysis

Data from the blood analysis and MWM task are presented as the mean ± standard deviation (SD). Data from the brain analysis are presented as the mean ± standard error of the mean (SEM). All data were analyzed using SPSS 18.0 software (SPSS, Chicago, IL, USA). Analysis of variance (ANOVA) with repeated measures was applied on the body weight in each week and the escape latency on each day; other data were analyzed with one-way ANOVA followed by Duncan’s post hoc test. A *p* < 0.05 was considered to indicate a significant difference.

## 3. Results

### 3.1. Blood Parameters and Body Weights

Serum glucose, TG, TC, HDL, LDL, and BW did not statistically differ among the three groups at the baseline (week 0, Table 1). During the first eight weeks the food intake was significantly greater in the experimental groups compared with the controls; however, no differences were observed between the two experimental groups (data not shown for brevity). After 20 weeks on the diets, the rats consuming the high-fructose-high-coconut oil diet had gained significantly less weight than those on the high-fructose-high-soybean oil diet (*p* < 0.05) (Table 2). However, no difference was found between the BWs of the high-fructose-high-coconut oil-fed and control rats, although the high-fructose-high-coconut oil-fed rats were slightly lighter (Table 2). The biochemical analyses performed in the 20th week of dietary intake showed that the rats fed the high-fructose-high-coconut oil diet had significantly higher glucose, TG, insulin, and HDL compared to the control group (all *p* < 0.05, Table 2). Although LDL had increased in rats receiving the high-fructose-high-coconut oil diet compared to the control and high-fructose-high-soybean oil-fed rats, this change did not reach a statistical difference (Table 2). Meanwhile, none of the serum parameters assayed differed between the soybean oil and control groups (*p* > 0.05, Table 2). Our observations reveal that the serum biochemical profiles of rats fed high-fructose-high-fat diets differed according to the type of fat. The coconut oil group exhibited a significantly higher adiposity index compared to the control and soybean oil groups (both *p* < 0.05, Table 2). The adiposity index was also significantly higher in the soybean oil group than in the control group (*p* < 0.05, Table 2).

### 3.2. Levels of Blood Fasting Glucose, Fructosamine and Leptin

**Figure 1:**
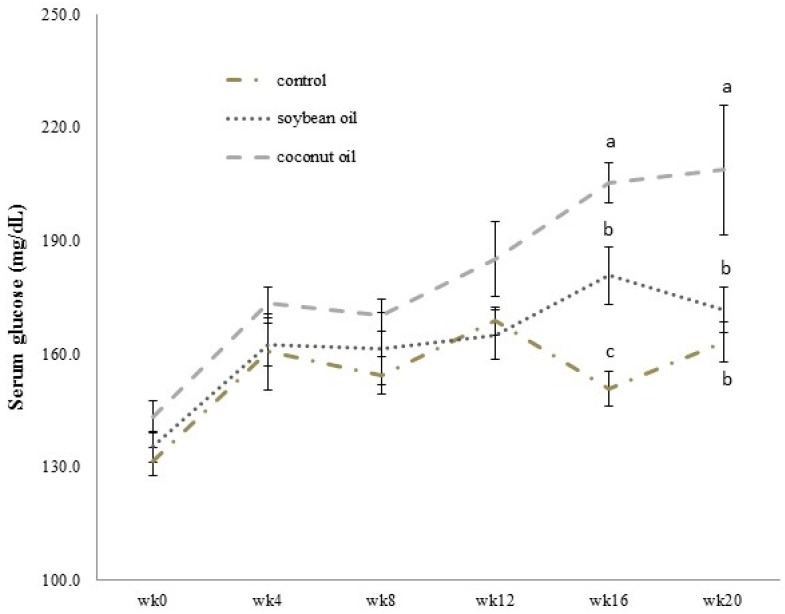
Changes in fasting serum glucose levels of rats from weeks 0 to 20 analyzed by ANOVA (with repeated measures each week). Data are shown as the mean ± SD of 8 (control)~12 (soybean oil and coconut oil) observations in each group. Within a week, groups with different letters are statistically significant to each other (*p* < 0.05).

**Figure 2:**
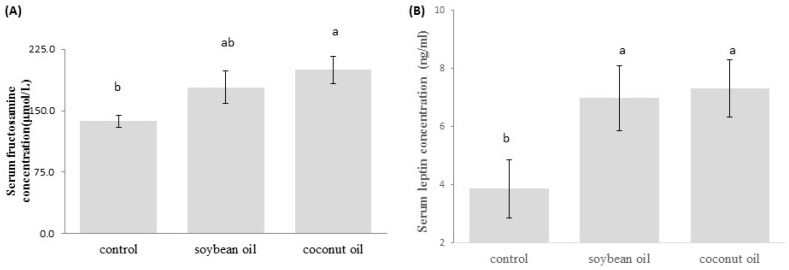
Levels of serum (**A**) fructosamine and (**B**) leptin in the control and experimental groups of rats at week 20. Data are the mean ± SD of 8 (control)~12 (soybean oil and coconut oil) observations and analyzed with one-way ANOVA Ducan’s post hoc analysis. Bars with different letters significantly differ (*p* < 0.05).

As shown in Figure 1, the fasting glucose blood level of the coconut oil group was generally higher and significantly increased starting at week 16 compared to animals on the high-fructose-high-soybean oil and control diets. However, in the soybean oil group the fasting glucose blood level was significantly higher than that in the control group at week 16, but returned to a similar level thereafter (*p* < 0.05, Figure 1). Rats receiving the high-fructose-high-coconut oil diet showed a significantly higher serum fructosamine level than did rats in the control and soybean oil groups (*p* < 0.05, Figure 2A), which indicated poor long-term glucose control in the high-fructose-high-coconut oil-fed rats. The serum leptin level in high-fructose-high-coconut oil-fed rats was significantly higher than that of the control rats (*p* < 0.05, Figure 2B). Although there was a tendency for the serum leptin level to be higher in the high-fructose-high-soybean oil-fed rats, this did not significantly differ between the groups (Figure 2B).

### 3.3. MWM Task for Spatial Learning and Memory

**Figure 3:**
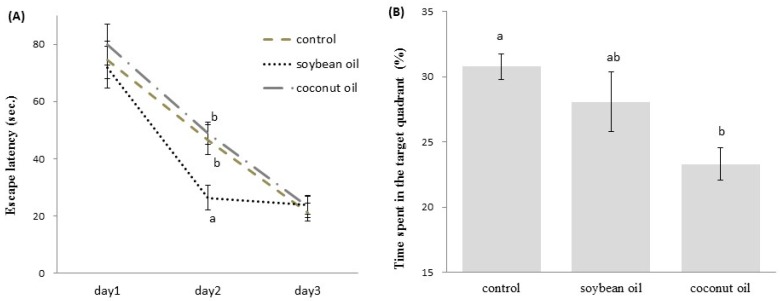
Spatial learning and memory training in the Morris water maze test of rats. Data are the mean ± SD of 8 (control)~12 (soybean oil and coconut oil) observations. (**A**) The latency of each rat to find a hidden platform during the three-day acquisition trials was analyzed by ANOVA with repeated measures. Within a day, groups with different letters are statistically significant to each other (*p* < 0.05); (**B**) Percentage of time spent in the target quadrant during a probe trial (day 4); data were analyzed with one-way ANOVA with Ducan’s post hoc analysis; bars with different letters significantly differ (*p* < 0.05).

The MWM task was employed to monitor the hippocampal-dependent learning and memory performance of the rats on different diets. During the training session, all groups of rats improved their performance as evidenced by the decreasing escape-latencies across successive days (Figure 3A). The coconut oil and control groups similarly learned the location of the hidden platform during the three days of training, indicating that the high-fructose-high-coconut oil diet had no effect on acquisition performance during training. However, the escape-latency was significantly lower in the high-fructose-high-soybean oil-fed rats than in the other two groups on the second day of training (*p* < 0.05, Figure 3A), indicating that the rats on the high-fructose-high-soybean oil diet performed better in spatial learning on day 2 of training. In the probe trial, the mean time that the high-fructose-high-coconut oil-fed rats spent in the target quadrant was significantly lower compared to the control or high-fructose-high-soybean oil-fed rats (both *p* < 0.05, Figure 3B). These findings suggest that 20 weeks of high-fructose-high-coconut oil consumption affected the hippocampal-dependent spatial memory behavior. In addition, there was a significant negative correlation between the performance in the probe trail of the MWM test and the serum TG level (coefficient *r* = −0.502, *p* = 0.0076).

### 3.4. Levels of Hippocampal Leptin, and mRNA Expressions of Leptin Receptor and SCD1

**Figure 4:**
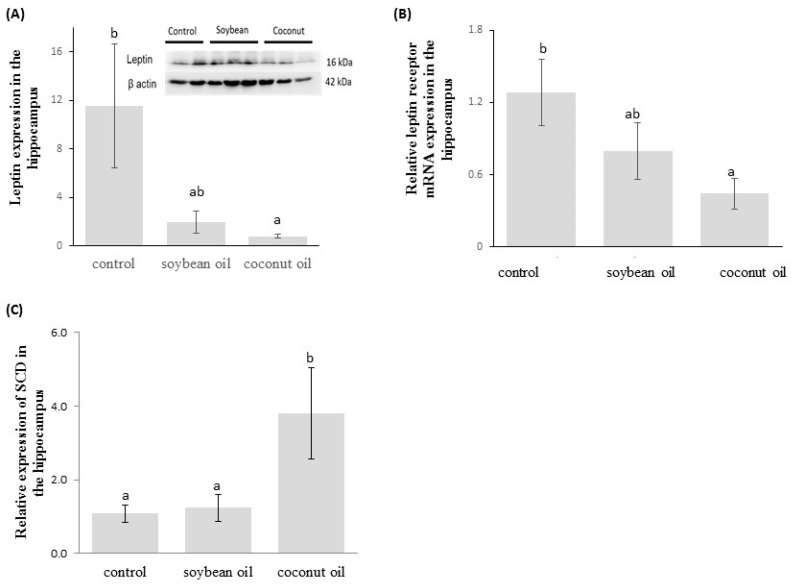
(**A**) Leptin protein expression; (**B**) Leptin receptor mRNA expression; and (**C**) Stearoyl-CoA desaturase (SCD) mRNA expression in the rat brain hippocampus after 20 weeks on the control or experimental diets. Data are expressed as the mean ± SEM (*n* = 3) and analyzed with one-way ANOVA with Ducan’s post hoc analysis. Bars with different letters significantly differ (*p* < 0.05).

As shown in Figure 4A (B), there was a significant reduction in leptin protein expression and leptin receptor mRNA expression in the hippocampus of the high-fructose-high-coconut oil-fed rats compared to the controls (*p* < 0.05). Rats fed the high-fructose-high-coconut oil diet had higher mRNA expression of SCD1 in the brain hippocampus than those fed either the control or high-fructose-high-soybean oil diets (*p* < 0.05, Figure 4C).

### 3.5. Fatty Acid Composition in the Brain

A profiling of various fatty acids in the brain in response to the diets was assayed by GC. The results of a detailed analysis of the brain fatty acid composition are shown in Table 3. In the brain, the contents of oleic acid (18:1, n-9), eicosenoic acid (20:1, n-9), arachidonic acid (AA, C20:4, n-6), and docosahexaenoic acid (DHA, C22:6, n-3) were significantly higher in the coconut oil group compared to the control and soybean groups (both *p* < 0.05, Table 3). The content of linoleic acid (C18:2, n-6) was significantly lower in the brain of the high-fructose-high-coconut oil-fed rats than that of the high-fructose-high-soybean oil-fed rats (*p* < 0.05, Table 3). Together, these results indicate that a high-fructose-high-coconut oil diet altered the fatty acid composition in the brain, whereas a high-fructose-high-soybean oil diet had no effect on it.

## 4. Discussion

It has been shown that the high-fructose-high-fat westernized diet is deleterious to metabolic homeostasis through its role in fatty livers, MetS, obesity, and diabetes [29], and that such diet-induced metabolic disorders may further impact mental health (including cognition) [30]. In this study, the rats were maintained on a high-fructose-high-fat diet for 20 weeks (based on a plant source of soybean or coconut oil) to demonstrate the effects of high-fructose-high-fat diets containing different types of dietary lipids on glucose homeostasis, lipid metabolism, peripheral leptin level, central leptin-mediated signaling, and hippocampal-dependent memory. Overall, these results suggest that a diet rich in saturated fatty acids is more effective in terms of producing metabolic disorders with concomitant changes in cognition than one rich in unsaturated fatty acids.

We observed that the high-fructose-high-coconut oil feeding in the present study caused a significant elevation in serum fructosamine, reflective of poor glycemic control over a short to medium period, and sustained hyperglycemia [31]. These findings suggest that under a high fructose dietary condition, saturated fat is more detrimental to glucose tolerance than unsaturated fat. It has been shown that the dietary component (saturated fat) may be responsible for the high-fat diet-induced disruption in glucose homeostasis [32,33]. Furthermore, the results show that the high-fructose-high-coconut oil diet led to significant elevation in circulating TG as well as relative total body fat in rats. It has been suggested that fat accumulation in the abdominal area is associated with the development of insulin resistance (independent of obesity) [34,35]. Thus, long-term consumption of a high-fructose-high-saturated fat (coconut oil) diet may predispose rats to hyperglycemia, hyperinsulinemia, and hypertriglyceridemia, all of which are components of MetS.

Regarding cognitive function, the current study demonstrates that hippocampal-dependent memory in rats was impaired after chronic feeding of the high-fructose-high-coconut oil diet as evidenced by poorer results in the MWM probe trial. This indicates that the memory retention ability of rats was affected by exposure to different types of fat (particularly saturated fats) when fructose was also present. Prevailing studies on the effects of a diet with a single nutrient component (i.e., either high fructose or high saturated fat) on cognitive function showed similar behavioral results [36,37]. These findings suggest that specific makers are needed to account for the concurrently observed, diet-induced, detrimental effect on hippocampal-dependent memory; such an effect must be attributed to other mechanisms (for instance, increased oxidative stress and the lower integrity of the blood-brain barrier (BBB) triggered by a diet high in fat are mediators of cognitive impairment [38]).

Leptin resistance in the brain is defined as a failure of leptin to cross the BBB [39]. It has been proposed that higher levels of circulating TG can limit leptin transport across the BBB in rodents [40]. As indicated above, our present data show that a high-saturated coconut oil diet caused significant elevations in serum TG and leptin, accompanied by significant reductions in the protein expression of leptin in the rat hippocampus. Collectively, it is plausible to speculate that chronic consumption of a high-fructose-high-saturated-fat (coconut oil) diet might lead to leptin resistance in the hippocampus. The assumption of leptin resistance might be further interpreted by the evidence of diminished hippocampal leptin protein and leptin receptor mRNA expressions due to the high-fructose-high-coconut-oil diet consumption, and that might be responsible for the impaired memory in the rat brains reported here. Based on these findings, we suggest that leptin plays a pivotal role in the impaired hippocampal-dependent memory elicited by our experimental paradigm. This suggestion is in agreement with previous studies emphasizing the role of leptin in hippocampal-dependent learning and memory [41]. It has also been shown that spatial memory impairment induced by a high-fat diet is associated with the brain leptin level and its relevant signal pathway [42]. This was also supported by other recent observations showing that low levels of leptin, leptin resistance, or leptin receptor deficiency in the hippocampus of diabetic rodents may be involved in cognitive deficits [43,44]. 

The regulation of the SCD1 gene was implicated in the development of diet-induced metabolic diseases [45]. As SCD1 is recognized as a lipogenic gene, it can be regulated by environmental factors such as dietary saturated fats or carbohydrates [46], and hormonal factors such as leptin [21]. The available evidence suggests that SCD1 is a molecular component of leptin signaling [21], and downregulation of SCD1 expression in the liver appears to be mediated by leptin signaling [47,48]. Moreover, leptin-deficient ob/ob mice exhibited high levels of SCD1 mRNA expression, suggesting the involvement of leptin in regulating the SCD1 gene [22]. The SCD1 gene encoding stearoyl-CoA desaturase is the rate-limiting enzyme in the biosynthesis of MUFAs [46]. Conversion of SFAs (i.e., palmitic and stearic acids) into MUFAs (palmitoleic and oleic acids) by stearoyl-CoA desaturase plays an important role in modifying the cell membrane fluidity and activating cell signaling pathways [48]. In this study, the hippocampal SCD1 mRNA levels in the rats fed the high-fructose-high-coconut oil diet increased as the hippocampal leptin levels and leptin receptor mRNA expression decreased. This might reflect increased synthesis of oleic acid as indicated by a higher amount of oleic acid in the brain, leading to alterations in the brain’s fatty acid composition (Table 3). In addition to increased oleic acid, we observed that the levels of AA and DHA were elevated in response to the high-fructose-high-coconut-oil diet consumption. The fatty acid profile in the brain has been indicated to be influenced by dietary or supplementary intake [49,50]. Although lower levels of AA and DHA in the lipid rafts on the membrane of neuron cells have been implicated in the occurrence of AD pathology [51], our results were acquired from the measurement of the whole brain rather from the lipid rafts alone. Furthermore, the animal model or the short feeding period in the present study might have affected the outcomes as well. 

## 5. Conclusions

Rats fed the high-fructose-high-coconut oil diet for 20 weeks were prone to develop hyperglycemia, hyperinsulinemia, hyperleptinemia, and hypertriglyceridemia. These metabolic consequences may contribute to hippocampal-dependent memory impairment. The mechanisms underlying the response of the memory function to the high-fructose-high- coconut oil diet might be relevant to the lower central leptin level and its molecular behavior in regulating the leptin signaling and SCD1 gene expression. 

## Figures and Tables

**Table 1:** Biochemical parameters and body weight (BW) in the control and experimental groups of rats at week 0.

Biochemical Parameters	Control	Soybean Oil	Coconut Oil
Glucose (mg/dL)	131.3 ± 4.36	135.3 ± 4.14	142.3 ± 4.72
Insulin (μg/L)	2.44 ± 0.12	2.58 ± 0.13	2.52 ± 0.17
TG (mg/dL)	75.9 ± 3.31	80.5 ± 7.77	78.6 ± 6.47
TC (mg/dL)	75.6 ± 3.31	78.3 ± 3.00	79.4 ± 1.77
HDL (mg/dL)	21.0 ± 0.97	21.2 ± 0.82	21.7 ± 1.07
LDL (mg/dL)	10.3 ± 1.23	9.42 ± 0.73	9.70 ± 0.52
BW (g)	260.6 ± 2.87	267.9 ± 2.05	267.2 ± 2.54

Data are expressed as the mean ± SD of 8 (control)~12 (soybean oil and coconut oil) observations in each group. Data were analyzed with one-way ANOVA (analysis of variance) with Ducan’s post hoc analysis. Above terms are defined as TG (triglyceride); TC (total cholesterol); HDL (high-density lipoprotein) and LDL (low-density lipoprotein).

**Table 2:** Biochemical parameters and body weight (BW) in the control and experimental groups of rats at week 20.

Biochemical Parameters	Control	Soybean Oil	Coconut Oil
Glucose (mg/dL)	159.8 ± 4.96 ^b^	169.1 ± 6.30 ^ab^	208.8 ± 17.2 ^a^
Insulin (μg/L)	1.29 ± 0.29 ^b^	1.88 ± 2.4 ^ab^	3.26 ± 0.44 ^a^
TG (mg/dL)	169.4 ± 23.9 ^b^	134.0 ± 11.3 ^c^	316.5 ± 20.6 ^a^
TC (mg/dL)	85.6 ± 4.44	75.7 ± 2.79	84.3 ± 2.89
HDL (mg/dL)	18.9 ± 1.90 ^b^	16.9 ± 0.48 ^c^	27.0 ± 0.97 ^a^
LDL (mg/dL)	6.14 ± 0.46	6.89 ± 0.39	7.27 ± 0.47
BW (g)	626.9 ± 26.12 ^ab^	662.9 ± 19.33 ^a^	604.6 ± 13.12 ^b^
Adiposity index ^#^ (%)	5.3 ± 0.8 ^c^	6.6 ± 0.5 ^b^	8.2 ± 0.9 ^a^

Data are expressed as the mean ± SD of 8 (control)~12 (soybean oil and coconut oil) observations in each group. Data were analyzed with one-way ANOVA with Ducan’s post hoc analysis. Within a row, numbers with different superscripts are statistically different at *p* < 0.05. TG (triglyceride); TC (total cholesterol); HDL (high-density lipoprotein) and LDL (low-density lipoprotein). ^#^ The adiposity index was calculated with the following equation: relative total body fat weight (%) = [the sum of the weight of abdominal and epididymal fat pads (g)/final body weight (g)] × 100%.

**Table 3:** Fatty acid composition of the brain in rats after 20 weeks on the control or experimental diets.

Fatty Acid (% of Total Fatty Acids)	Control	Soybean Oil	Coconut Oil
Myristic acid (C14:0)	0.65 ± 0.09	0.54 ± 0.14	0.72 ± 0.10
Palmitic acid (C16:0)	5.54 ± 1.19	4.23 ± 1.35	5.19 ± 2.09
Palmitoleic acid (C16:1)	1.58 ± 1.51	4.47 ± 1.59	3.75 ± 1.64
Stearic acid (C18:0)	1.52 ± 1.09	0.98 ± 0.61	0.27 ± 0.08
Oleic acid (C18:1)	6.51 ± 0.70 ^b^	6.22 ± 0.39 ^b^	8.98 ± 1.03 ^a^
Linoleic acid (C18:2)	1.94 ± 1.00 ^b^	4.48 ± 1.50 ^a^	1.00 ± 0.70 ^b^
Linolenic acid (C18:3)	0.14 ± 0.04	0.33 ± 0.12	0.18 ± 0.07
Eicosenoic acid (C20:1)	0.26 ± 0.06 ^b^	0.25 ± 0.07 ^b^	0.70 ± 0.13 ^a^
Eicosadienoic acid (C20:2)	1.31 ± 0.61	1.57 ± 0.55	0.61 ± 0.45
Arachidonic acid (C20:4)	2.37 ± 0.30 ^b^	2.31 ± 0.46 ^b^	4.10 ± 0.73 ^a^
Docosapentaenoic acid (C22:5)	1.59 ± 0.76	1.57 ± 0.64	0.54 ± 0.49
Docosahexaenoic acid (C22:6)	2.69 ± 0.33 ^b^	2.35 ± 0.49 ^b^	4.25 ± 0.69 ^a^

Data are expressed as the mean ± SD of 8 (control)~12 (soybean oil and coconut oil) observations in each group. Data were analyzed with one-way ANOVA with Ducan’s post hoc analysis. Within a row, numbers with different superscripts are statistically different at *p* < 0.05.

## References

[1] Phillips C.M. (2013). Nutrigenetics and metabolic disease: Current status and implications for personalised nutrition. Nutrients.

[2] Walker J.M., Harrison F.E. (2015). Shared neuropathological characteristics of obesity, type 2 diabetes and alzheimer’s disease: Impacts on cognitive decline. Nutrients.

[3] Solfrizzi V., Scafato E., Capurso C., D’Introno A., Colacicco A.M., Frisardi V., Vendemiale G., Baldereschi M., Crepaldi G., Di Carlo A. (2011). Metabolic syndrome, mild cognitive impairment, and progression to dementia. The italian longitudinal study on aging. Neurobiol. Aging.

[4] Morris M.C., Tangney C.C. (2014). Dietary fat composition and dementia risk. Neurobiol. Aging.

[5] Francis H., Stevenson R. (2013). The longer-term impacts of western diet on human cognition and the brain. Appetite.

[6] Kanoski S.E., Davidson T.L. (2011). Western diet consumption and cognitive impairment: Links to hippocampal dysfunction and obesity. Physiol. Behav..

[7] Winocur G., Greenwood C.E. (2005). Studies of the effects of high fat diets on cognitive function in a rat model. Neurobiol. Aging.

[8] Pistell P.J., Morrison C.D., Gupta S., Knight A.G., Keller J.N., Ingram D.K., Bruce-Keller A.J. (2010). Cognitive impairment following high fat diet consumption is associated with brain inflammation. J. Neuroimmunol..

[9] Lakhan S.E., Kirchgessner A. (2013). The emerging role of dietary fructose in obesity and cognitive decline. Nutr. J..

[10] Hsu T.M., Konanur V.R., Taing L., Usui R., Kayser B.D., Goran M.I., Kanoski S.E. (2015). Effects of sucrose and high fructose corn syrup consumption on spatial memory function and hippocampal neuroinflammation in adolescent rats. Hippocampus.

[11] Lozano I., Van der Werf R., Bietiger W., Seyfritz E., Peronet C., Pinget M., Jeandidier N., Maillard E., Marchioni E., Sigrist S. (2016). High-fructose and high-fat diet-induced disorders in rats: Impact on diabetes risk, hepatic and vascular complications. Nutr. Metab..

[12] Dearborn J.L., Knopman D., Sharrett A.R., Schneider A.L., Jack C.R., Coker L.H., Alonso A., Selvin E., Mosley T.H., Wagenknecht L.E. (2014). The metabolic syndrome and cognitive decline in the atherosclerosis risk in communities study (aric). Dement. Geriatr. Cogn. Disord..

[13] Crescenzo R., Bianco F., Coppola P., Mazzoli A., Cigliano L., Liverini G., Iossa S. (2015). The effect of high-fat--high-fructose diet on skeletal muscle mitochondrial energetics in adult rats. Eur. J. Nutr..

[14] Huang H.Y., Korivi M., Tsai C.H., Yang J.H., Tsai Y.C. (2013). Supplementation of lactobacillus plantarum k68 and fruit-vegetable ferment along with high fat-fructose diet attenuates metabolic syndrome in rats with insulin resistance. Evid. Based Complement. Altern. Med..

[15] Luo Y., Burrington C.M., Graff E.C., Zhang J., Judd R.L., Suksaranjit P., Kaewpoowat Q., Davenport S.K., O’Neill A.M., Greene M.W. (2016). Metabolic phenotype and adipose and liver features in a high-fat western diet-induced mouse model of obesity-linked nafld. Am. J. Physiol. Endocrinol. Metab..

[16] Manrique C., DeMarco V.G., Aroor A.R., Mugerfeld I., Garro M., Habibi J., Hayden M.R., Sowers J.R. (2013). Obesity and insulin resistance induce early development of diastolic dysfunction in young female mice fed a western diet. Endocrinology.

[17] Panchal S.K., Poudyal H., Iyer A., Nazer R., Alam M.A., Diwan V., Kauter K., Sernia C., Campbell F., Ward L. (2011). High-carbohydrate, high-fat diet-induced metabolic syndrome and cardiovascular remodeling in rats. J. Cardiovasc. Pharmacol..

[18] Shapiro A., Tumer N., Gao Y., Cheng K.Y., Scarpace P.J. (2011). Prevention and reversal of diet-induced leptin resistance with a sugar-free diet despite high fat content. Br. J. Nutr..

[19] Bonda D.J., Stone J.G., Torres S.L., Siedlak S.L., Perry G., Kryscio R., Jicha G., Casadesus G., Smith M.A., Zhu X. (2014). Dysregulation of leptin signaling in alzheimer disease: Evidence for neuronal leptin resistance. J. Neurochem..

[20] Zhang Y., Proenca R., Maffei M., Barone M., Leopold L., Friedman J.M. (1994). Positional cloning of the mouse obese gene and its human homologue. Nature.

[21] Ntambi J.M., Miyazaki M. (2003). Recent insights into stearoyl-coa desaturase-1. Curr. Opin. Lipidol..

[22] Miyazaki M., Kim Y.C., Ntambi J.M. (2001). A lipogenic diet in mice with a disruption of the stearoyl-coa desaturase 1 gene reveals a stringent requirement of endogenous monounsaturated fatty acids for triglyceride synthesis. J. Lip. Res..

[23] Cohen P., Miyazaki M., Socci N.D., Hagge-Greenberg A., Liedtke W., Soukas A.A., Sharma R., Hudgins L.C., Ntambi J.M., Friedman J.M. (2002). Role for stearoyl-coa desaturase-1 in leptin-mediated weight loss. Science.

[24] Astarita G., Jung K.M., Vasilevko V., Dipatrizio N.V., Martin S.K., Cribbs D.H., Head E., Cotman C.W., Piomelli D. (2011). Elevated stearoyl-coa desaturase in brains of patients with alzheimer’s disease. PLoS ONE.

[25] Morris R. (1984). Developments of a water-maze procedure for studying spatial learning in the rat. J. Neurosci. Methods.

[26] Vorhees C.V., Williams M.T. (2006). Morris water maze: Procedures for assessing spatial and related forms of learning and memory. Nat. Protoc..

[27] Madison D.V., Edson E.B. (2001). Preparation of hippocampal brain slices. Curr. Protoc. Neurosci..

[28] Folch J., Lees M., Sloane Stanley G.H. (1957). A simple method for the isolation and purification of total lipides from animal tissues. J. Biol. Chem..

[29] Parafati M., Lascala A., Morittu V.M., Trimboli F., Rizzuto A., Brunelli E., Coscarelli F., Costa N., Britti D., Ehrlich J. (2015). Bergamot polyphenol fraction prevents nonalcoholic fatty liver disease via stimulation of lipophagy in cafeteria diet-induced rat model of metabolic syndrome. J. Nutr. Biochem..

[30] Agrawal R., Gomez-Pinilla F. (2012). ‘Metabolic syndrome’ in the brain: Deficiency in omega-3 fatty acid exacerbates dysfunctions in insulin receptor signalling and cognition. J. Physiol..

[31] Armbruster D.A. (1987). Fructosamine: Structure, analysis, and clinical usefulness. Clin. Chem..

[32] von Frankenberg A.D., Marina A., Song X., Callahan H.S., Kratz M., Utzschneider K.M. (2017). A high-fat, high-saturated fat diet decreases insulin sensitivity without changing intra-abdominal fat in weight-stable overweight and obese adults. Eur. J. Nutr..

[33] Crescenzo R., Bianco F., Coppola P., Mazzoli A., Tussellino M., Carotenuto R., Liverini G., Iossa S. (2014). Fructose supplementation worsens the deleterious effects of short-term high-fat feeding on hepatic steatosis and lipid metabolism in adult rats. Exp. Physiol..

[34] Ruderman N., Chisholm D., Pi-Sunyer X., Schneider S. (1998). The metabolically obese, normal-weight individual revisited. Diabetes.

[35] Seppala-Lindroos A., Vehkavaara S., Hakkinen A.M., Goto T., Westerbacka J., Sovijarvi A., Halavaara J., Yki-Jarvinen H. (2002). Fat accumulation in the liver is associated with defects in insulin suppression of glucose production and serum free fatty acids independent of obesity in normal men. J. Clin. Endocrinol. Metab..

[36] Boitard C., Cavaroc A., Sauvant J., Aubert A., Castanon N., Laye S., Ferreira G. (2014). Impairment of hippocampal-dependent memory induced by juvenile high-fat diet intake is associated with enhanced hippocampal inflammation in rats. Brain Behav. Immun..

[37] Ross A.P., Bartness T.J., Mielke J.G., Parent M.B. (2009). A high fructose diet impairs spatial memory in male rats. Neurobiol. Learn. Mem..

[38] Freeman L.R., Haley-Zitlin V., Rosenberger D.S., Granholm A.-C. (2014). Damaging effects of a high-fat diet to the brain and cognition: A review of proposed mechanisms. Nutr. Neurosci..

[39] Haring S.J., Harris R.B. (2011). The relation between dietary fructose, dietary fat and leptin responsiveness in rats. Physiol. Behav..

[40] Banks W.A. (2012). Role of the blood-brain barrier in the evolution of feeding and cognition. Ann. N. Y. Acad. Sci..

[41] Farr S.A., Banks W.A., Morley J.E. (2006). Effects of leptin on memory processing. Peptides.

[42] Valladolid-Acebes I., Fole A., Martin M., Morales L., Cano M.V., Ruiz-Gayo M., Del Olmo N. (2013). Spatial memory impairment and changes in hippocampal morphology are triggered by high-fat diets in adolescent mice. Is there a role of leptin?. Neurobiol. Learn. Mem..

[43] Yang C., Zhu B., Hua F. (2014). Leptin deficiency is involved in the cognitive impairment of streptozocin-induced diabetic rats undergoing cardiopulmonary bypass. Int. J. Clin. Exp. Med..

[44] Li X.L., Aou S., Oomura Y., Hori N., Fukunaga K., Hori T. (2002). Impairment of long-term potentiation and spatial memory in leptin receptor-deficient rodents. Neuroscience.

[45] Park E.I., Paisley E.A., Mangian H.J., Swartz D.A., Wu M.X., O’Morchoe P.J., Behr S.R., Visek W.J., Kaput J. (1997). Lipid level and type alter stearoyl coa desaturase mrna abundance differently in mice with distinct susceptibilities to diet-influenced diseases. J. Nutr..

[46] Ntambi J.M. (1995). The regulation of stearoyl-coa desaturase (scd). Prog. Lip. Res..

[47] Mauvoisin D., Prevost M., Ducheix S., Arnaud M.P., Mounier C. (2010). Key role of the erk1/2 mapk pathway in the transcriptional regulation of the stearoyl-coa desaturase (scd1) gene expression in response to leptin. Mol. Cell. Endocrinol..

[48] Biddinger S.B., Miyazaki M., Boucher J., Ntambi J.M., Kahn C.R. (2006). Leptin suppresses stearoyl-coa desaturase 1 by mechanisms independent of insulin and sterol regulatory element-binding protein-1c. Diabetes.

[49] Jicha G.A., Markesbery W.R. (2010). Omega-3 fatty acids: Potential role in the management of early alzheimer’s disease. Clin. Interv. Aging.

[50] Amtul Z., Westaway D., Cechetto D.F., Rozmahel R.F. (2011). Oleic acid ameliorates amyloidosis in cellular and mouse models of alzheimer’s disease. Brain Pathol..

[51] Martin V., Fabelo N., Santpere G., Puig B., Marin R., Ferrer I., Diaz M. (2010). Lipid alterations in lipid rafts from alzheimer’s disease human brain cortex. J. Alzheimer’s Dis..

